# Neutrophil cannibalism – a back up when the macrophage clearance system is insufficient

**DOI:** 10.1186/1465-9921-7-143

**Published:** 2006-12-14

**Authors:** Kristina Rydell-Törmänen, Lena Uller, Jonas S Erjefält

**Affiliations:** 1 Div. Vascular and Airway Research, Dept. Experimental Medical Science, Lund University, Lund, Sweden

## Abstract

**Background:**

During a lipopolysaccharide-induced lung inflammation, a massive accumulation of neutrophils occurs, which is normally cleared by macrophage phagocytosis following neutrophil apoptosis. However, in cases of extensive apoptosis the normal clearance system may fail, resulting in extensive neutrophil secondary necrosis. The aim of this study was to explore the hypothesis that neutrophils, in areas of the lung with extensive cellular infiltration, contribute to clearance by phagocytosing apoptotic cells and/or cell debris derived from secondary necrosis.

**Methods:**

Intranasal lipopolysaccharide administration was used to induce lung inflammation in mice. The animals were sacrificed at seven time points following administration, bronchoalveolar lavage was performed and tissue samples obtained. Electron microscopy and histochemistry was used to assess neutrophil phagocytosis.

**Results:**

Electron microscopic studies revealed that phagocytosing neutrophils was common, at 24 h after LPS administration almost 50% of the total number of neutrophils contained phagosomes, and the engulfed material was mainly derived from other neutrophils. Histochemistry on bronchoalvolar lavage cells further showed phagocytosing neutrophils to be frequently occurring.

**Conclusion:**

Neutrophils are previously known to phagocytose invading pathogens and harmful particles. However, this study demonstrates that neutrophils are also able to engulf apoptotic neutrophils or cell debris resulting from secondary necrosis of neutrophils. Neutrophils may thereby contribute to clearance and resolution of inflammation, thus acting as a back up system in situations when the macrophage clearance system is insufficient and/or overwhelmed.

## Background

Neutrophils are short lived immune cells who invade tissues in response to a variety of stimuli, for example viral and bacterial infections [1,2]. They are professional phagocytes and contribute to resolution of inflammation by removing infectious and inflammatory stimuli [1,2]. Apart from being present during acute infections, neutrophils are also found to a variable degree during airway diseases such as COPD, asthma and ARDS/ALI [3,4]. Neutrophils have a high turnover and are normally rapidly cleared by apoptosis, followed by macrophage phagocytosis [2,5]. During infection a large number of neutrophils are present in order to efficiently clear the infection, and studies have shown that ingestion of bacteria may delay neutrophil apoptosis [2], thereby causing very large number of cells accumulating in the same area. In such cases, the normally rapid clearance mechanisms are even more necessary, since vast numbers of neutrophils pose a serious threat to the surrounding tissue. If the apoptotic neutrophils are not cleared away efficiently or fast enough they undergo secondary necrosis, which is a pro-inflammatory event [6]. Using an animal model of lipopolysaccharide (LPS)-induced inflammation, we have previously demonstrated extensive neutrophil infiltration followed by apoptosis and secondary necrosis of neutrophils in areas of intense inflammation and neutrophil infiltration (inflammatory foci, IF) [7]. Interestingly, in IF we found apparently viable neutrophils with phagosomes enclosing what appeared to be whole apoptotic neutrophils, apoptotic nuclei and other neutrophil cell remnants. The aim of the present study was to prove the existence of this phenomenon and quantify its occurrence through detailed ultrastructural studies, and test the hypothesis that neutrophils contribute to clearance in localized areas where the macrophage system is insufficient. We frequently found phagocytosing neutrophils in IF and BALF, with phagosomes of varying size containing what appeared to be whole apoptotic neutrophils, apoptotic nuclei and neutrophil-derived cell debris. Phagocytosing macrophages were present in both IF and in BALF but in IF, the macrophage clearance system seemed to be insufficient (indicated by the large number of neutrophils undergoing secondary necrosis) and in addition, several macrophages in IF displayed signs of necrosis.

Previously, neutrophils phagocytosing apoptotic cells and nuclei have been described in blood smears from patients with systemic lupus erythematosus (SLE), a feature called LE cells [8-10]. However, to our knowledge phagocytosing neutrophils has not been described *in vivo *or in lungs before. Areas similar to the foci investigated in our study are present during pneumonias [11,12], and most likely also during COPD exacerbations and ALI/ARDS. Due to the pro-inflammatory effect of secondary necrosis [13,14], we suggest that neutrophils in IF may contribute to resolution of inflammation by phagocytosing apoptotic neutrophils and/or neutrophil-derived cell debris. This study thus assigns neutrophils a hitherto unknown role, namely to contribute to resolution of inflammation by phagocytosis of cell debris derived from neutrophils.

## Methods

### Animals

Female Balb/c mice, 6–8 weeks old were obtained from MoB A/S (Ry, Denmark). All protocols were approved by the local ethics committee (Malmö/Lund, Sweden).

### LPS-Induced lung inflammation

A total dose of 50 μg LPS (*E. coli*, Sigma, St Louis, MO, USA), was administered intranasally during light anaesthesia as previously described [7]. BAL were performed as previously described [15] and tissue samples were obtained for paraffin (H&E) and plastic embedding (electron microscopy) [15]. Total and differential cell counts in BALF were obtained using a haemocytometer and May-Grünewald/Giemsa-stained cytospin slides. The presence of an inflammatory response was determined by cellular infiltration into the lung parenchyma (H&E) and increased numbers of immune cells in BALF. The activity of the cytoplasm enzyme lactatedehydrogenase (LDH) in lavage fluid was used as a pan-necrosis marker. The content of LDH was enzymatically determined in 100 μl BALF, by the Laboratory of Clinical Chemistry, Lund University Hospital, Lund, Sweden, as previously described [7].

### Phagocytosis by BALF macrophages and neutrophils

DNA-positive phagosomes in BALF neutrophils and macrophages was visualized on cytospin slides by the general DNA marker Hoechst 33342 (20 mg/ml, Sigma) and analyzed by fluorescence microscopy. DNA-positive phagosomes were clearly visible as characteristic blue dots in the cytoplasm of the phagocyte. The proportion of phagocyte-positive neutrophils and macrophages was calculated for each time point and compared to controls.

### Transmission Electron Microscopy (TEM)

TEM analysis was performed as described elsewhere [7,16]. The IF were subjected to a detailed ultrastructural analysis (as previously described in [7]), and the involvement of neutrophils in the clearance process was studied by assessing the number of phagocytosing neutrophils. For each time point 3 areas were studied, at least 90 neutrophils counted (with the exception of controls, where neutrophils were very scarce), and the proportion of phagocyting neutrophils was calculated and compared to control. At 36 h after LPS administration, when the number of neutrophils in BALF peaked, an extended analysis on phagocyte-containing neutrophils was conducted. The number of granulae per area (μm^2^) cytoplasm in neutrophils with and without large phagosomes was calculated on electron microscopic photomicrographs.

### Statistical analysis

For calculations, independent sample t-test was employed and all groups were compared against control, using the statistic program Analyze It™ (Analyze-it Software Ltd, Leeds, UK). Data are given as mean values ± SEM, and p ≤ 0.05 was considered statistically significant.

## Results

### LPS induces lung inflammation

**Figure 1:**
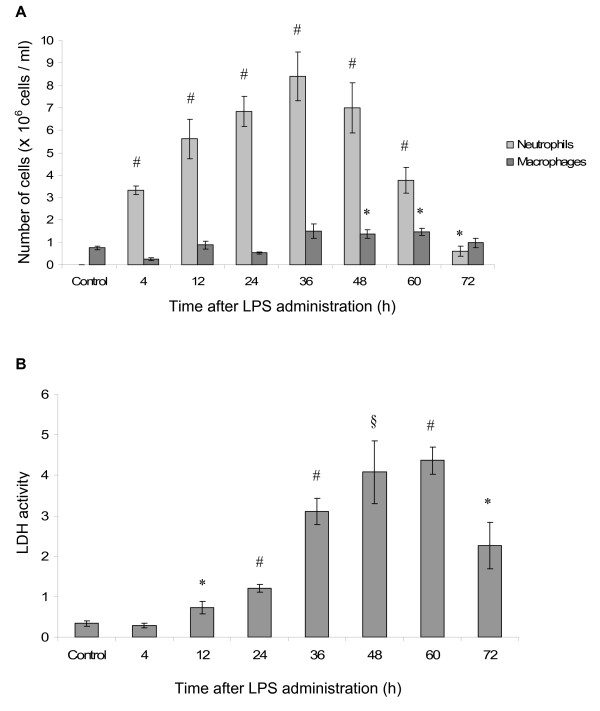
**LPS induces lung inflammation**. The number of neutrophils and macrophages in BALF increased significantly in response to LPS (**A**), both peaking at 36 h after administration. The lavage content of lactatedehydrogenase (LDH) also increased in response to LPS (**B**), and reached maximum levels 60 h after LPS administration. The data are given as mean ± SEM and compared against control using independent samples t-test. * indicates p < 0.05, § indicates p < 0.01 and # indicates p < 0.001.

The presence of an inflammatory response was determined by increased numbers of neutrophils and macrophages in BALF (Figure 1A), and the activity of the pan-necrosis marker LDH (Figure 1B). In the tissue, a patchy neutrophil-rich inflammatory pattern was confirmed by histological (H&E) analysis of the lung parenchyma (for a closer description, see [7]).

### Neutrophil phagocytosis in inflammatory foci

**Figure 2:**
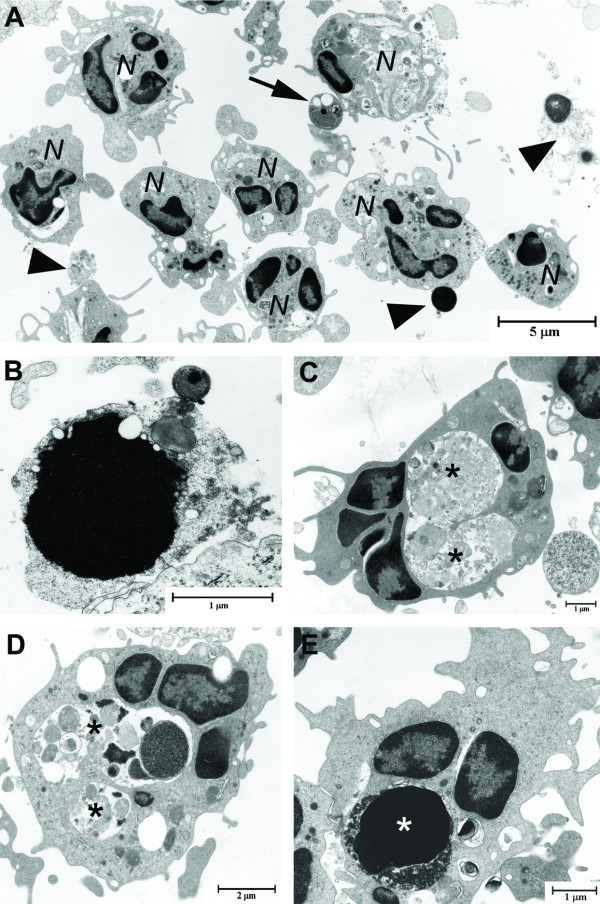
**Representative transmission electron micrographs displaying neutrophils during an LPS-induced lung inflammation**. In areas of intense inflammation and neutrophil infiltration highly activated neutrophils (*N*), characterized by e.g. phagosomes and/or cytoplasmatic protrusions, were lying amongst apoptotic neutrophils (black arrow) and cell debris (black arrowhead) (**A**). Also secondary necrosis (characterized by membrane rupture of cells with an otherwise apoptotic morphology) of neutrophils was regularly observed (**B**). Furthermore, neutrophils containing large phagosomes (asterisks) enclosing neutrophilic cell remnants such as apoptotic nuclei and neutrophil granulae (**C-E**) were frequently found.

**Figure 3:**
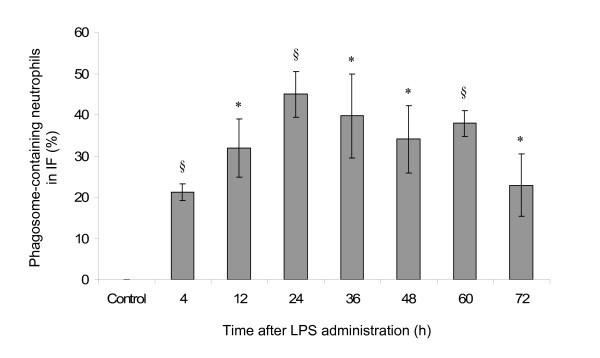
**The proportion of phagocytosing neutrophils in inflammatory foci (IF) varied between different time points**. The data are given as mean percentages ± SEM and compared against control using independent samples t-test. * indicates p < 0.05 and § indicates p < 0.01.

Determined by TEM, the neutrophils found inside the alveolar wall or the subepithelial tissue surrounding bronchi and bronchioles displayed generally little or no signs of activation. In contrast, alveolar luminal neutrophils were generally activated; both apoptosis and secondary necrosis (Figure 2A), as well as extracellular neutrophil granules, free condensed nuclei and other types of cell debris was regularly seen (Figure 2B), primarily at 24 h and onwards. Phagocytosing neutrophils were frequently found (Figure 2C–E), the numbers significantly increasing already 4 h after LPS administration, peaking at 24 h (Figure 3). When assessing the granulae content in neutrophils with large phagosomes 36 h after LPS administration, a significant decrease in the number of granulae was detected, (0.21 ± 0.07 granulae/μm^2^) compared to neutrophils without phagosomes (1.5 ± 0.37 granulae/μm^2^). We also noted that the phagosome content varied over time, from 24 h and onwards phagosomes generally contained cell remnants (e.g. apoptotic nuclei and neutrophilic granulae), and at the earlier time points mainly surfactant.

### Neutrophil phagocytosis in BALF

**Figure 4:**
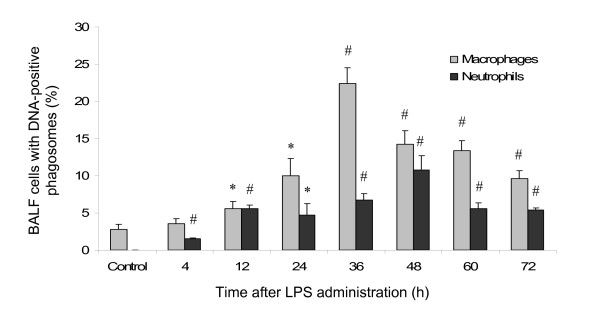
**The proportion of phagocyting macrophages (MQ) and neutrophils (PMN) in lavage fluid varied between the different time points**. The numbers are given as percentage of total number of cells (macrophages or neutrophils), expressed as mean percentages ± SEM and compared against control using independent samples t-test. * indicates p < 0.05 and # indicates p < 0.001.

**Figure 5:**
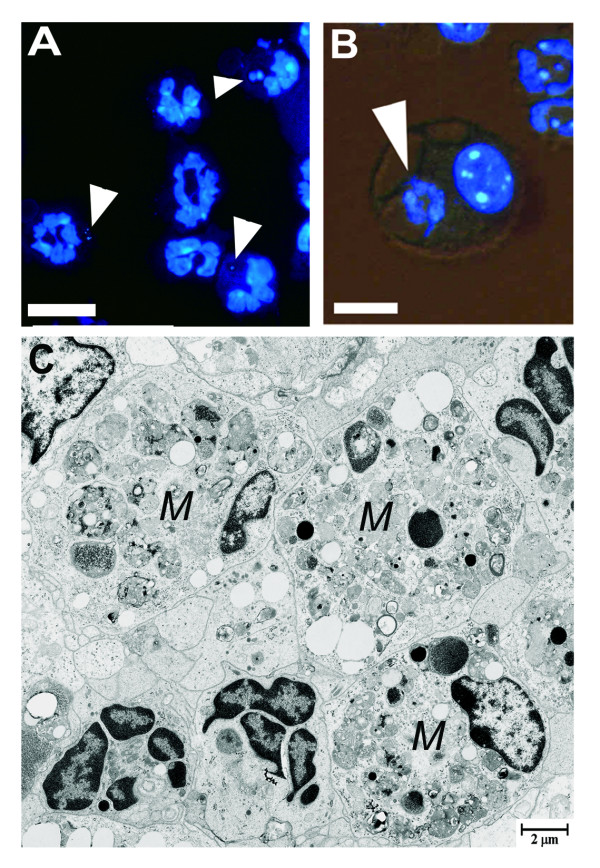
**Phagocytosing neutrophils and macrophages in lavage fluid**. Photomicrographs illustrating BALF neutrophils (**A**) and macrophages (**B**) containing DNA-positive phagosomes (indicated by white arrowheads). DNA was visualized by labelling with the fluorescent membrane permeable DNA-marker Hoechst 33342. Scale bars indicate 10 μm in A and 5 μm in B. In areas of intense inflammation and neutrophil infiltration, a majority of the macrophages (*M*) were abundantly packed with multiple large phagosomes (**C**).

Also in BALF, neutrophils containing phagosomes were found (Figure 4), detected as DNA-positive phagosomes in neutrophils (Figure 5A). The number of phagocytosing neutrophils increased significantly following LPS administration and peaked at 48 h after LPS administration when 10.8 ± 2% of the BALF neutrophils contained DNA-positive phagosomes. In control animals, none of the exceedingly rare neutrophils contained any DNA-positive phagosomes.

### Macrophage phagocytosis

The number of macrophages in BALF containing DNA-positive phagosomes increased after LPS administration, peaking at 36 h (Figure 4 and 5B). A vast majority of the alveolar macrophages in IF contained abundant phagosomes with whole apoptotic cells or cell debris (Figure 5C). In IF, scattered macrophages also displayed signs of necrosis, revealed by chromatolytic nucleus and electron lucent cytoplasm.

## Discussion

It is previously well known that neutrophils contribute to resolution of inflammation and clearance of pathogens during infection by killing and phagocyting pathogens. In the present study, using a model of LPS-induced lung inflammation we propose yet another mechanism by which they contribute to the resolution of inflammation: by phagocytosing apoptotic cells and/or cell remnants.

Neutrophils are classified as professional phagocytes, and are important in resolution and clearance of pathogens [1,2]. They are known to phagocytose pathogens (including yeast and bacteria) as well as potentially hazardous substances, being a fist line defence [17]. Normally neutrophils die through apoptosis, followed by subsequent macrophage phagocytosis. However, if macrophages fail to clear the apoptotic neutrophils, apoptotic neutrophils are left in the tissue and undergoes secondary necrosis [6]. The model used in our study produces a patchy inflammation with foci of extensive cellular infiltration, inflammatory foci or IF [7]. In IF, neutrophils with phagosomes containing both what appeared to be whole apoptotic neutrophils as well as apoptotic nuclei and neutrophil derived cell debris, were frequently found. Besides neutrophils, phagocytosing macrophages were present in both IF and BALF, but in IF, the macrophage clearance system seemed to be insufficient to meet the needs (indicated by the large number of apoptotic cells, mainly neutrophils, in the process of secondary necrosis) and in addition, several macrophages in IF displayed signs of necrosis. Phagocytosing neutrophils were also found in BALF, but at lower numbers. However, the results obtained in BALF only include cells with DNA-positive phagosomes, whereas the electron microscopic study of IF includes all cells with phagosomes, suggesting the BALF-values to be falsely low.

Unfortunately, we could not determine whether neutrophils (either from BALF or IF) had phagocytosed intact apoptotic cells, or only cell remnants of secondary necrosis, i.e. free condensed nuclei, neutrophil granulae and other cell components. However, in several cases (see for example figure 2E), it did look as if whole apoptotic neutrophils were ingested. The phagosome content varied between time points, reflecting the inflammatory situation: At early time points the phagosomes were small and contained mainly surfactant, whereas at later time points the contents was ranging from what appeared to be whole apoptotic neutrophils to apoptotic nuclei and gatherings of neutrophil granulae. Furthermore, we found that neutrophils containing large phagosomes contained less granulae. We cannot rule this out as an artefact; however, it suggests that neutrophils lose at least a part of their granulae before or during phagocytosis. This implies that attempts to identify neutrophils via labelling of their granulae proteins may prove unsuccessful in situations where neutrophils are engaged in phagocytosis.

Unlike macrophages which are known to phagocytose apoptotic or necrotic cells as well as cell debris, neutrophils have to our knowledge never been ascribed this capacity. The only previous descriptions, depict an *ex vivo *feature of Systemic Lupus Erythematosus (SLE), called "LE cells" [9,10,18,19]. LE cells appear in blood smears from patients with SLE, and within the smears, phagocytic cells with large phagosomes can be seen. Schmidt-Acevedo *et. al*. [9] concluded that the LE cell phenomenon represents non-professional phagocytosis of apoptotic bodies. Furthermore neutrophils have been described to phagocytose dead cells or cell nuclei [18] and are known to phagocytose erythrocytes [20]. However, to our knowledge neutrophils phagocytosing cell remnants during a lung inflammation has never been described before.

Several phagocytic signals, for example phosphatidylserine (PS) expression on the surface of apoptotic cells, and apoptosis receptors including CD14, as well as lectin, scavenger and Fc-receptors [21,22] are known to be critically involved in the process of recognition and engulfment. These receptors are expressed on the cell surface of macrophages, but are interestingly also found on neutrophils [23-25], suggesting neutrophils to have a similar phagocytic capacity as macrophages.

It is apparent that neutrophils have the abilities needed to mimic macrophage behaviour; they attend the site of inflammation or infection, have clearance/phagocytosis capacity and are present in large numbers in areas where the macrophage system appears to be insufficient. Furthermore, the number of phagocytosing macrophages peaked 12 h before the number of phagocytosing neutrophils, suggesting that neutrophil phagocytosis is a stage proceeding macrophage phagocytosis. All together, this suggests that neutrophils may in fact contribute to the clearance and resolution of an inflammation by removing pro-inflammatory cell debris from the tissue, thereby acting as a back up system stepping in when the macrophage system is exhausted. This suggestion is supported by a study exploring the effects of ozone on airway epithelial cells *in vitro *[26], were the authors reported neutrophils to enhance the removal of ozone injured epithelial cells, facilitating repair of the epithelial cell layer. Based on this, we suggest that neutrophils may in fact be beneficial to inflammatory resolution during certain inflammatory conditions.

From our results, it seems clear that neutrophils phagocytosing cell remnants are not a common phenomenon, but occurs in somewhat extreme situations, such as in IF when/if the macrophage clearance system is exhausted, which may explain why this phenomenon had not been described before. In IF, an extensive infiltration of neutrophils results in large numbers of apoptotic cells which likely overwhelm the macrophage clearance system (which is satiable) locally and result in secondary necrosis. However, a similar extreme situation might occur during a more moderate neutrophil infiltration, if the macrophage system is impaired, for example due to problems with recognition and/or clearance of the apoptotic cells. A risk of impairment has been shown in several studies, for example in macrophages exposed to smoke or collected from COPD patients [27,28], and LPS stimulated alveolar macrophages from patients suffering from severe asthma [29]. This suggests that phagocytosing neutrophils may occur during several clinical conditions. From the present study, it can be concluded that the most likely site for clearance failure, are in areas of intense inflammation and cellular infiltration. Such areas frequently occur during e.g. common lung infections [11,12], and probably also during COPD exacerbations and ARDS/ALI. The prevalence of neutrophil phagocytosis in clinical situations is currently unclear, likely due to the facts that no one (to our knowledge) has actively studied this feature before, the patchy occurrence of IF and the difficulties to obtain samples from the lung parenchyma of living patients. An important task will now be to confirm the present findings in relevant human material, and characterize the process thoroughly.

## Conclusion

In summary, we report that neutrophils can phagocytose apoptotic neutrophil remnants and most likely whole apoptotic neutrophils as well, thereby assigning them a never before described function in lungs. The exact mechanisms behind the phagocytosis of apoptotic neutrophils is currently unknown, but neutrophils do express most, if not all, surface receptors used by macrophages in the process of phagocytosis, suggesting the mechanisms to be similar in the two cell types. Based on our findings in mice we suggest that neutrophil phagocytosis of apoptotic neutrophils and/or neutrophilic cell remnants (neutrophil cannibalism) may be relatively commonly occurring in situations of dense neutrophil infiltration. These situations include the inflammatory foci investigated in our study, and most likely also clinical conditions such as infectious pneumonias, ARDS/ALI and COPD-exacerbations. However, to gain certainty and further knowledge, additional studies in animal models as well as in clinical situations, are now highly warranted.

## Competing interests

The author(s) declare that they have no competing interests.

## Authors' contributions

KRT participated in the design of the study, played a major role in the acquisition, analysis and interpretation of data, and drafted the manuscript. LU participated in the *in vivo*-procedures, analysis of the data and writing the manuscript. JSE participated in the design of the study, the *in vivo*-procedures and writing of the manuscript. All authors read and approved the final manuscript.
